# Quantifying biased technical progress in China: Heterogeneous human capital and labor force dynamics

**DOI:** 10.1016/j.heliyon.2024.e33540

**Published:** 2024-06-29

**Authors:** Xiuli Cui, Ehsan Elahi, Bo Xu, Jiaxun Xing, Mohd Shuaib, Zainab Khalid

**Affiliations:** a School of Economics, Shandong University of Technology, Zibo, 255049, China; b School of Economics and Social Welfare, Zhejiang Shuren University, Hangzhou, China; c Chongqing Zhihai Electronic Technology Co., Ltd, China; d Department of Mechanical Engineering, Delhi Technological University, Delhi, India; e School of Economics and Management, Southeast University, Nanjing, 210096, China

**Keywords:** Human capital, Labor heterogeneity, Elasticity of substitution, Technical progress, China

## Abstract

The main objectives of this study are to estimate the biased technical progress in China considering heterogeneity in human capital and labor force, examine how this heterogeneity quantitatively impacts the elasticity of substitution and biased technical progress compared to homogeneous labor assumptions, and compare the growth rates of human capital-augmenting and labor-augmenting technical progress. The estimation procedure involves a constrained Seemingly Unrelated Regression (SUR) using provincial-level panel data from 1985 to 2021. By comparing results with and without accounting for labor force heterogeneity and human capital, the analysis quantifies their impact on the estimated elasticity of substitution between factors and the magnitude and direction of technical bias. Results found that the elasticity of capital-labor (human capital) substitution is between 0.7 and 0.8, and the elements are generally complementary. Although it does not affect the overall trend of elasticity of substitution and biased technical progress, the heterogeneity of human capital and labor force has a quantitative impact on them. It means that the elasticity of substitution increased, and the biased technological progress decreased. Moreover, the growth rate of human capital-augmenting technical progress was significantly lower than that of labor-augmenting technological progress. This study estimates the biased technical progress in China, where the human capital and labor force are heterogeneous. The findings suggest that policymakers should prioritize human capital investments and technological upgrades in industries to rebalance China's technical progress and boost productivity growth.

## 1 Introduction

Biased technical progress has been studied for a long time [1,2]. As early as the era of classical economics, scholars were already concerned about the technological progress in economic growth [3,4]. They also noted that technical progress is unbalanced in improving production factors' efficiency and saving their input. Marx's theory of the organic composition of capital can be regarded as the early expression of biased technical progress. Hicks formally put forward this concept in The Theory of Wages in 1932. Since then, the theoretical exploration and empirical analysis of biased technological progress has entered a comprehensive and in-depth stage.

Foreign-related research started earlier; there was theoretical and empirical research on biased technical progress. Fellner [5] and Kennedy [6] measured the biased technological progress of Western developed countries, especially the United States. Samuelson [7] constructed a simplified model of capital-augmenting or labor-augmenting technical progress based on their research. Berndt [8] conducted an empirical analysis around the elasticity of substitution, one of the core variables affecting biased technological progress. These studies laid a foundation for further research on biased technical progress, but generally, they were based on neoclassical economic growth theory. Endogenous growth theory, which emerged in the 1980s, held that endogenous technological progress is the decisive factor of economic growth. Under the framework of this theory, Daren Acemoglu [[9], [10], [11], [12]] revealed the determinants and mechanism of technical innovation direction of enterprises and explained the theoretical basis of biased technological progress from the micro level of enterprises. Qiu et al. [13] used a data envelopment approach to estimate the technological innovation efficiency of medical manufacturing firms. Klump et al. [14], Ryuzo Sato [15] and Sato and Morita [15] proposed a new measurement method of biased technical progress.

Domestic research started relatively late, and most focused on the measurement and impact analysis of biased technical progress. Lu and Zhang [16], Lei [17], Shi-chuan [18], Huang and Xu [19], Dai [2] and Wu et al. [20] measured the elasticity of factor substitution and biased technical progress from different angles. More scholars have focused on the impact of biased technological progress. For instance, Zhang et al. [21], Han [22] and Yang and Ouyang [23] empirically analyzed the effect of biased technical progress on labor income share. Yuan and Ouyang [23] examined the influence of technological progress on total factor productivity. Feng et al. [24], and Zhang et al. [25] demonstrated the effects of technical progress on employment, economic growth, and energy efficiency.

The concept of biased technical progress has a long history in economic thought. In classical economics, scholars recognized that technological progress did not uniformly enhance the productivity of all factors of production [9]. Marx's theory of the rising organic composition of capital highlighted the tendency of technical change to favor capital over labor [11]. Shove [26] coined the formal term "biased technical progress" to describe this phenomenon.

Empirical studies in the mid-20th century found evidence of biased technical progress in developed countries. Fellner [5] and Kennedy [6] identified capital-augmenting technical change in the United States and other Western economies. Samuelson [7] developed a simplified model of factor-augmenting technical progress based on this work. Berndt [8] further investigated the elasticity of substitution between capital and labor, a key parameter shaping biased technical change.

Later theoretical work sought to endogenize the direction of technical change. Under the induced innovation hypothesis, the relative scarcity and prices of factors of production steer the development of new technologies to conserve the scarce factor [6,7,26]. In the endogenous growth literature, Acemoglu [9] found the direction of technical change based on firms' profit-maximizing innovation decisions. The elasticity of substitution and relative factor prices determine the factor bias of innovations. Similarly, Li et al. [27] found a relationship between job stress and innovation ideas.

On the measurement front, Klump et al. [14] and Sato & Morita [15] advanced new empirical approaches to estimate biased technical change using normalized supply-side systems and flexible functional forms. Previous studies applied these methods to China and found evidence of capital-augmenting technical progress [15,17,20]. However, most of this work assumed homogeneous labor inputs. Zhang et al. [4] and Zhang et al. [28] emphasized the labor skills heterogeneity to estimate the labor mismatch in industrial enterprises.

The role of human capital in economic growth is well-established. Schultz [29] pioneered the theory of human capital, arguing that investments in education and skills drive productivity growth. Nelson and Phelps [30] proposed that human capital facilitates technology adoption and diffusion. A large empirical literature confirms human capital's positive effect on growth [[31], [32], [33]].

However, fewer studies examine human capital's impact on the direction of technical change. Acemoglu [9] developed a theory in which the relative supply of skilled labor induces skill-biased technical change. Studies by Autor et al. [34] and Acemoglu and Autor [35] found that the college wage premium and share of skilled labor have risen over time in the U.S., consistent with skill-biased technical change. In a growth accounting framework, Vandenbussche et al. [36] found that human capital composition affects the skill bias of productivity growth.

For China, studies have documented the importance of human capital to economic growth [27,[37], [38], [39], [40], [41], [42]]. Some scholars have also noted the heterogeneity in educational attainment across Chinese regions [27,38,39,[43], [44], [45]]. However, there is limited research on how this heterogeneity affects the direction of technical progress in China.

These studies have found interesting results, but the common ground of these studies is the homogenization of production factors. It can be considered nearly accurate in developed countries such as the United States. In the developed countries, the monetized material capital can be considered homogeneous. However, in China, the homogenization of the labor force does not exist. By 2021, people with a college degree or above accounted for only 23.2 % of the total population. At the same time, the population in Hong Kong and Taiwan with this education level accounted for nearly 40 % and 52.6 %, respectively. Regarding high school, the proportion was 44.0 %, 74 %, and 86.7 %,^2^ respectively. The education level in the Chinese Mainland was relatively low. However, relevant studies have shown that human capital positively impacts economic growth, which will change the production function of biased technical progress. In other words, the production function with the human capital factor is significantly different from that without this factor. This difference will affect the biased technical progress. Therefore, this study analyzes the biased technical progress founded on labor heterogeneity and human capital and compares it with the labor homogenization situation to explore the influence of human capital and labor heterogeneity on biased technical progress.

This study aims to address the research gap by 1) Analyzing biased technical progress in China based on a production function incorporating labor heterogeneity and human capital, 2) Quantifying how human capital and labor force heterogeneity impact the elasticity of substitution and biased technical progress compared to homogeneous labor assumptions and 3) Comparing the growth rates of human capital-augmenting and labor-augmenting technical progress. The rationale is that achieving these objectives will provide a more accurate understanding of the nature of China's biased technical progress and the role of human capital. The potential contributions include 1) Yielding new insights into China's technical progress by considering important heterogeneity in labor, 2) Showing the quantitative effects of labor force composition on measures of factor substitutability and technical bias and 3) Clarifying the relative importance of human capital versus raw labor in China's technical progress. These findings could offer valuable policy implications. For example, they may suggest increasing human capital investments and technology intensity as feasible approaches to adjust China's technical progress and improve factor productivity. By providing a clearer and more realistic picture of China's biased technical change and human capital's role, this study aims to support evidence-based policymaking to guide China's future economic growth and development.

## 2 Materials and methods

According to Lei [17], we conduct the theoretical analysis and estimation method of biased technology progress by using two factors' Constant Elasticity of Substitution (CES) production function.

### 2.1 Measurement method of biased technical progress without human capital

We used the profit maximization method to derive the calculation of biased technical progress. Assuming that the enterprise is the main body of social production and operation, its goal is to maximize profits. Besides labor and capital, the enterprise needs to consider the existence of government departments in the operation process, so the government tax should be considered. These are the wage range of labor ($w_{t}$), capita rent rate ($r_{t}$), and government tax rate ($\tau_{t}$). The enterprise profits are total output deduct capital costs, labor costs, and government taxes. The specific formulae are given in equations (1), (2), (3).

(1) $$\pi_{t}=F\left(A_{t}K_{t},B_{t}L_{t}\right)-r_{t}K_{t}-w_{t}L_{t}-\tau_{t}F\left(A_{t}K_{t},B_{t}L_{t}\right)$$

By taking derivatives of K and L, the first derivative conditions of profit-maximizing incentives are:

(2) $$\frac{\partial \pi_{t}}{\partial K_{t}}=\left(1-\lambda_{t}\right)F_{K}-r_{t}=0$$

(3) $$\frac{\partial \pi_{t}}{\partial L_{t}}=\left(1-\lambda_{t}\right)F_{L}-w_{t}=0$$

This shows that in the presence of government tax, capital and labor return rates are their respective marginal outputs after deducting the production tax rate.

The output per effective worker is $\tilde{q}=Q/BL$, and the capital per effective worker is $\tilde{k}=AK/BL$; therefore, the production function is provided in equation (4).

(4) $$\tilde{q}=f\left(\tilde{k}\right)=F\left(AK/BL,1\right)$$

Giving $s_{t}=1-\tau_{t}$, the return on capital and the wage rate are r and w, respectively. Their mathematical forms are given in equations (5), (6)).

(5) $$r=sAf^{\prime }\left(\tilde{k}\right)$$

(6) $$w=s^{\prime }B\left[f\left(\tilde{k}\right)-\tilde{k}f^{\prime }\left(\tilde{k}\right)\right]$$

The elasticity of input factor substitution in production is σ, and the capital-labor ratio is k=K/L; the marginal product of the labor-capital ratio is $p=F_{L}/F_{K}$. It can be written as in equation (7).

(7) $$\sigma =\frac{\partial \ln\;k}{\partial \ln\;p}=-\frac{d\left(K/L\right)}{d\left(F_{K}/F_{L}\right)}/\frac{K/L}{F_{K}/F_{L}}$$

Provided that the production function is homogeneous of the first degree with twice continuous differentiability, according to Euler theorem. It can be written as in equation (8).

(8) $$\sigma =\frac{F_{K}F_{L}}{FF_{KL}}$$

From equations (4), (8)), we can deduce the elasticity of substitution of capital and labor, and equation (9) can be written as:

(9) $$\sigma =-\frac{f^{\prime }\left(\tilde{k}\right)\left[f\left(\tilde{k}\right)-\tilde{k}f^{\prime }\left(\tilde{k}\right)\right]}{\tilde{k}f^{\prime }\left(\tilde{k}\right)f^{\prime \prime }\left(\tilde{k}\right)}$$

The wage rate per effective worker is $\tilde{w}=w/B$, combined with equation (6). Therefore, equation (10) can be written as:

(10) $$\frac{\tilde{w}}{s}=f\left(\tilde{k}\right)-\tilde{k}f^{\prime }\left(\tilde{k}\right)$$

Equation (11) is derived from equation (10) after taking the derivative of $\left(\tilde{w}/s\right)$.

(11) $$1=f^{\prime }\frac{d\tilde{k}}{d\tilde{q}}\frac{d\tilde{q}}{d\left(\tilde{w}-s\right)}-\tilde{k}f^{\prime \prime }\frac{d\tilde{k}}{d\tilde{q}}\frac{d\tilde{q}}{d\left(\tilde{w}/s\right)}-f^{\prime }\frac{d\tilde{k}}{d\tilde{q}}\frac{d\tilde{q}}{d\left(\tilde{w}/s\right)}$$

From Equation (4) we can write Equation (12).

(12) $$\frac{d\tilde{k}}{d\tilde{q}}=\frac{1}{f^{\prime }}$$

Therefore, we can write Equation (13) as:

(13) $$\frac{d\tilde{q}}{d\left(\tilde{w}/s\right)}=-\frac{f^{\prime }}{kf^{\prime \prime }}$$

From equations (4), (10), (13), (9), we can deduce the elasticity of factor substitution, and equation (14) can be written as:

(14) $$\sigma =\frac{d\tilde{q}}{d\left(\tilde{w}/s\right)}\frac{\tilde{w}/s}{\tilde{q}}$$

Regarding the output per worker $q=Q/L\tilde{q}=q/B$ and the theory of logarithmic derivative, we can derive another expression σ from equation (12). Therefore, equation (15) can be written as:

(15) $$\sigma =\frac{\frac{\dot{q}}{q}-\frac{\dot{B}}{B}}{\frac{\dot{w}}{w}-\frac{\dot{s}}{s}-\frac{\dot{B}}{B}}$$

Then the labor-augmenting technical progress rate can be written as in equation (16).

(16) $$\frac{\dot{B}}{B}=\frac{\frac{\dot{q}}{q}-\sigma \left(\frac{\dot{w}}{w}-\frac{\dot{s}}{s}\right)}{1-\sigma },\sigma \neq 1$$

Similarly, given the unit capital output z=Q/K, the capital-augmenting technical progress rate can be written as in equation (17).

(17) $$\frac{\dot{A}}{A}=\frac{\frac{\dot{z}}{z}-\sigma \left(\frac{\dot{r}}{r}-\frac{\dot{s}}{s}\right)}{1-\sigma },\sigma \neq 1$$

In terms of Hicks [46] definition of biased technical progress, Diamond [47] inferred the biased technical progress index, which can be written as in equation (18).

(18) $$D=-\frac{\partial \ln\;p}{\partial t}=\frac{F_{K_{t}}}{F_{K}}-\frac{F_{L_{t}}}{F_{L}}$$

where $F_{K_{t}}=\partial F_{K}/\partial t$ represents the increment of marginal product of capital and $F_{L_{t}}=\partial F_{L}/\partial t$ represents the increment of marginal product of labor, which are brought about by technical progress. The definition of a biased technical progress index is the former minus the latter. If D>0, the technical progress is biased toward capital. But if D<0, the technical progress is biased toward labor. Moreover, if D=0, these two growth rates are equal, which is called the Hicks-neutral technical progress.

If technical progress is exogenous, capital-augmenting technology index A and labor-augmenting technology index B are both functions of time T. The calculation formula of D can be derived as in equation (19).

(19) $$D=\left(1-\frac{1}{\sigma }\right)\left(\frac{\dot{A}}{A}-\frac{\dot{B}}{B}\right)$$

The biased technical progress depends on the elasticity of factor substitution σ, and the rate of labor- and capital-augmenting technological progress.

### 2.2 Estimation of the elasticity of factor substitution

To measure the degree of technical progress, we must first estimate the elasticity of factor substitution σ and set the production function in the form of CES. It can be presented in equation (20).

(20) $$Q={\left[\theta {\left(A_{t}K_{t}\right)}^{-\rho }+\left(1-\theta \right){\left(B_{t}L_{t}\right)}^{-\rho }\right]}^{-\frac{1}{\rho }}$$

ρ=(1−σ)/σ≥−1

where θ∈(0,1) is the factor allocation parameter, ρ is the substitution parameter σ∈(0,∞) and it is the elasticity of factor substitution.

Based on equation (18), we can calculate the marginal product of capital and labor using equations (21), (22)), respectively.

(21) $$F_{K}=\frac{\partial Q_{t}}{\partial K_{t}}=\theta {A_{t}}^{\left(\sigma -1\right)/\sigma }{\left(\frac{Q_{t}}{K_{t}}\right)}^{\frac{1}{\sigma }}$$

(22) $$F_{L}=\frac{\partial Q_{t}}{\partial L_{t}}=\left(1-\theta \right){B_{t}}^{\left(\sigma -1\right)/\sigma }{\left(\frac{Q_{t}}{L_{t}}\right)}^{\frac{1}{\sigma }}$$

By substitute equation (19), (2), (20), (3), equations (23), (24)) can be written as:

(23) $$r_{t}=\theta \left(1-\tau_{t}\right){A_{t}}^{\left(\sigma -1\right)/\sigma }{\left(\frac{Q_{t}}{K_{t}}\right)}^{\frac{1}{\sigma }}$$

(24) $$r_{t}=\left(1-\theta \right)\left(1-\tau_{t}\right){B_{t}}^{\left(\sigma -1\right)/\sigma }{\left(\frac{Q_{t}}{L_{t}}\right)}^{\frac{1}{\sigma }}$$

Since capital-augmenting technical level $A_{t}$ and labor-augmenting technical level $B_{t}$ cannot be observed we assume that they change separately as exponential form to estimate.

We put $A_{t}=A_{0}e^{\gamma K^{t}+\varepsilon K_{t}}$, and $B_{t}=B_{0}e^{\gamma L^{t}+\varepsilon L_{t}}$ into (21), (22), then take the logarithm to this function to derive equations (25), (26)).

(25) $$\ln\left(\frac{Q_{t}}{K_{t}}\right)=\ln\left(\theta^{-\sigma }{A_{0}}^{1-\sigma }\right)+\gamma_{K}\left(1-\sigma \right)t+\sigma \;\ln\left(\frac{r_{t}}{1-\tau_{t}}\right)+\left(1-\sigma \right)\varepsilon_{K_{t}}$$

(26) $$\ln\left(\frac{Q_{t}}{L_{t}}\right)=\ln\left[{\left(1-\theta \right)}^{-\sigma }{B_{0}}^{1-\sigma }\right]+\gamma_{L}\left(1-\sigma \right)t+\sigma \;\ln\left(\frac{w_{t}}{1-\tau_{t}}\right)+\left(1-\sigma \right)\varepsilon_{L_{t}}$$

where $\gamma_{K}$ is the average growth rate of capital-augmenting technical level; $\gamma_{L}$ is the average growth rate of labor-augmenting technical level. $\varepsilon_{K_{t}}$ and $\varepsilon_{L_{t}}$ are random interferences, which are period-dependent. Hence, the constrained system estimation method should be evaluated using equations (23), (24)). Therefore, Equations (27), (28)) can be written as.

(27) $$y_{K_{t}}=\ln\left(\frac{Q_{t}}{K_{t}}\right),y_{L_{t}}=\ln\left(\frac{Q_{t}}{L_{t}}\right)$$

(28) $$x_{K_{t}}=\ln\left(\frac{r_{t}}{1-\tau_{t}}\right),x_{L_{t}}=\ln\left(\frac{w_{t}}{1-\tau_{t}}\right)$$

Similarly, Equations (25), (26)) can be simplified and written as Equations (29), (30)).

(29) $$y_{K_{t}}=\delta_{K}+\eta_{K}t+\sigma x_{K_{t}}+\mu_{K_{t}}$$

(30) $$y_{L_{t}}=\delta_{L}+\eta_{L}t+\sigma x_{L_{t}}+\mu_{L_{t}}$$

For observation values of n period time-series sample, we divided the explanatory and explanatory variables in equations (27), (28) vector y of 2n dimension and matrix X of 2n×5 dimension. It can be presented in Equation (31).

(31) $$y=\left(\begin{array}{c} y_{K}\\ y_{L} \end{array}\right),X=\left(\begin{array}{ccccc} i & t & 0 & 0 & x_{K}\\ 0 & 0 & i & t & x_{L} \end{array}\right)$$

where i is the n-dimension vector with all values of 1, and 0 is the dimension with 0 vector. At the same time, the coefficients in equations (27), (28)) are arranged into vectors. It can be presented in Equation (32).

(32) $$\varphi ={\left(\begin{array}{ccccc} \delta_{K} & \eta_{K} & \delta_{K} & \eta_{L} & \sigma \end{array}\right)}^{\prime }$$

By combining the sample observation equations (29), (30)), equation (33) can be written as:

(33) y=Xφ+u

where $u={\left({u_{K}}^{\prime }{u_{L}}^{\prime }\right)}^{\prime }$ is 2n dimension stochastic impact vector, while the covariance matrix of stochastic impact vector in period t is $u_{t}={\left({u_{K_{t}}}^{\prime }{u_{L_{t}}}^{\prime }\right)}^{\prime }$. Similarly, Equation (34) can be written as:

(34) $$E\left(u_{t}{u_{t}}^{\prime }\right)=\sum =\left(\begin{array}{cc} \sigma_{K}^{2} & \sigma_{KL}\\ \sigma_{LK} & \sigma_{L}^{2} \end{array}\right)$$

The covariance matrix of stochastic impact vector u can be written as in equation (35).

(35) $$E\left(u_{t}u_{t}^{\prime }\right)=\Omega =\sum ⊗I_{n}$$

By using the Generalized Least Squares (GLS) method to estimate equation (31), we can get the estimated value of the regression coefficient vector φ (Equation (36)).

(36) $$\hat{\varphi }={\left(X^{\prime }\left(\sum^{-1}⊗I\right)X\right)}^{-1}X^{\prime }\left(\sum^{-1}⊗I\right)y$$

Usually, the covariance matrix ∑ of the stochastic impact vector is unknown. Firstly, we use the ordinary least square method to calculate the estimation value $\hat{\sum }$. Afterward, we applied the Feasible Generalized Least Squares (FGLS) method, to estimate the regression coefficient vector of the model.

### 2.3 Measurement of biased technical progress considering human capital

There are many ways to measure human capital, including the education period method, labor remuneration method, and education investment method, among others. The calculation formula is given in Equation (37).

(37) $$H_{i}=\sum_{i=1}^{6}EH_{i}h_{i}$$

where $EH_{i}$ refers to the number of people at the education level i, and $h_{i}$ is the average education years at education level i. There is a lack of official data on the education years. Using official and research data, the China Center for Human Capital and Labor Market Research of the Central University of Finance and Economics divided education level into six categories.^3^ Then the number of people with various education levels was estimated. These people, aged from 0 to 65, come from different provinces and cities in mainland China, Hong Kong, and Taiwan. They were divided into urban and rural areas, gender, and education level.^4^ Combining the education levels of the population from 16 to 65 in the mainland provinces (excluding Hainan and Tibet, where most of the data required for the estimation model is missing, and incorporating the Chongqing data into Sichuan), we can obtain the average number of education years of the labor force. It can be used to substitute the middle education years of the employees approximately, then multiply it by the average number of people in each province. We can estimate the total number of each section $H_{i}$. Use H to replace L in formulas (from 1 to 37) to get the estimation method of biased technical progress index and elasticity of substitution considering human capital.

## 3 Empirical estimation of factor elasticity of substitution and biased technical progress

As mentioned above, the estimated equations without considering human capital are equations (29), (8)). When considering human capital, equation (29) remains unchanged, and Equation (38) can be derived from Equation (30).

(38) $$yH_{t}=\delta H+\eta H_{t}+\sigma XH_{t}+\mu H_{t}$$

$$yH_{t}=\ln\frac{Q_{t}}{H_{t}},XH_{t}=\ln\left(\frac{\omega H_{t}}{1-\tau_{t}}\right)$$

where $\omega H_{t}$ is the return rate of human capital, as mentioned above $\omega_{t}$.

Therefore, the results can be obtained by using the constrained SUR estimates for the simultaneous system equations composed of equations (29), (30)), and equations (29), (36)).

### 3.1 Data source

In 1984, China started to implement the reform of the economic system. Therefore, we chose 1985–2021 to calculate the degree of biased technical progress. The data we need is as follows.

$Q_{t}$: Gross output of the social finished product is due to the GDP in the statistical yearbook of each province. Then we converted it into the real GDP value of 1978 as the base year and tooled it as the total output sequence.
$K_{t}$: Capital quantity. The National Bureau of Statistics did not calculate and release the capital quantity in the production process. Jun et al. [48] found the real stock of fixed capital. Moreover, the data from 1985 to 2021 was estimated according to their method from 2001 to 2021.
$L_{t}$: Quantity of labor force is the number of employees at the end of each year, published in the statistical yearbook of each province.
$H_{t}$: Quantity of human capital is as mentioned earlier. Unit: 10 thousand people a year.
$r_{t}$: Return on capital. It is expressed by the sum of the actual depreciation of fixed assets and the actual operating surplus divided by the actual fixed capital investment.
$w_{t}$: The return rate of labor is calculated by dividing the total amount of actual labor remuneration by the number of employees.
$\omega H_{t}$: The return rate of human capital is calculated by dividing the total amount of actual labor remuneration by the amount of human capital.
$\tau_{t}$: The government's production tax rate is calculated as net real production tax divided by real GDP.

In 1992, the direction of the socialist market up the GDP accounting data items calculated by the provincial income method in each year, we got the data of labor remuneration, depreciation of fixed assets, net production tax, and an operating surplus in the initial distribution of finished products from 1985 to 2021, then converted them into the actual value of 1978, as the base year.

## 4 Results and discussion

During China's reform process, several key historical events significantly impacted economic development: the formal establishment of the socialist market economy reform direction in 1992, China's accession to the WTO in 2001, and the global financial crisis in 2008. Therefore, three dummy variables, dum92, dum02, and dum08, reflect these historical events. These variables are defined in Equations (39), (40), (41).

(39) $$dum92=\left\{ \begin{array}{c} 0,1985-1991\\ 1,1992-2021 \end{array}\right.$$

(40) $$dum02=\left\{ \begin{array}{c} 0,1985-2001\\ 1,2002-2021 \end{array}\right.$$

(41) $$dum08=\left\{ \begin{array}{c} 0,1985-2007\\ 1,2008-2021 \end{array}\right.$$

These three dummy variables are added to the above two groups of estimation equations. The specific estimation results are shown in Table 1. The value of R^2^ provides the goodness of fit of a statistical model [49]. The values of R^2^ in all models are almost 0.99, it depicts that 99 % of changes in the dependent variables are due to explained variables that are included in the models and 0.01 % variation is due to those variables that are not included in the models.

**Table 1:** Constrained SUR regression results of elasticity of factor substitution and biased technical progress at the national level.

Variables	Model 1		Model 2	
	$\ln\frac{GDP_{t}}{K_{t}}$	$\ln\frac{GDP_{t}}{L_{t}}$	$\ln\frac{GDP_{t}}{K_{t}}$	$\ln\frac{GDP_{t}}{H_{t}}$
Constant	5.813	−21.65**	4.497	−6.260
	(1.02)	(–3.10)	(0.80)	(–1.17)
σ	0.732***	0.732***	0.766***	0.766***
	(13.52)	(13.48)	(14.48)	(14.48)
t	−0.0027	0.0112**	−0.0021	0.0032
	(-0.94)	(3.02)	(–0.66)	(1.11)
dum92	20.21***	−34.50***	18.76**	−34.54***
	(3.36)	(–4.85)	(3.28)	(–4.56)
dum92*t	−0.0102**	0.0178***	−0.0994**	0.0180***
	(–3.35)	(4.85)	(–3.28)	(4.56)
dum02	34.61***	−51.75***	39.53***	−55.84***
	(3.89)	(–7.37)	(4.29)	(–7.97)
dum02*t	−0.0187***	0.0272***	−0.0195***	0.0278***
	(3.89)	(7.37)	(4.29)	(7.98)
dum08	−30.42**	67.69***	−36.40**	70.39***
	(–2.46)	(9.29)	(–2.97)	(8.91)
dum08*t	0.0217*	−0.0409***	0.0191**	−0.0315***
	(2.47)	(–9.29)	(2.98)	(–8.92)
R^2^	0.9962	0.9998	0.9960	0.9996

***, ** and * represent significance levels at 1 %, 5 %, and 1 %, respectively. The given values in the parentheses are the t-statistics.

The results of Table 2 depict that the Chinese labor-capital substitution elasticity ranges from 0.7 to 0.8, and the two elements are complementary in general. The capital efficiency-related parameters are negative, while the labor (human capital) efficiency-related parameters are positive. It demonstrated that from 1985 to 2021, Chinese capital production efficiency declined while labor productivity (human capital) improved. It can also be found that after considering human capital, the elasticity of substitution increases steadily, and the influence of human capital on other parameters has no apparent direction.

**Table 2:** Various biased technical progress indices in different periods from 1985 to 2021.

Period	Without human capital			With human capital		
	$\gamma_{K}$	$\gamma_{L}$	D	$\gamma_{K}$	$\gamma_{H}$	D
1985–1991	−0.00964	0.040876	0.019063	−0.00802	0.012675	0.006645
1992–2001	−0.04686	0.105839	0.057631	−0.04893	0.086749	0.043554
2002–2007	−0.11182	0.201825	0.118375	−0.12877	0.205267	0.107226
2008–2021	−0.05552	0.076465	0.049314	−0.05578	0.062374	0.037385

In light of the regression results and definition of each parameter, we can get the results of the average rate of capital-augmenting technical progress $\gamma_{K}$, the average rate of labor-augmenting technical progress $\gamma_{L}$, the average rate of human capital-augmenting technical progress $\gamma_{H}$, and the index of biased technological progress in different periods from 1985 to 2021. The results are shown in Table 1.

Table 2 shows that the average rate of capital-augmenting technical progress, the average rate of labor-augmenting technological progress, and the average rate of human capital-augmenting technical progress confirm the previous analysis. The index of biased technical progress is positive, indicating that technical progress belongs to the capital-biased type, which is consistent with the conclusion of most scholars.

After considering the influence of human capital, the absolute value of all indices is lower than that without human capital, but the direction does not change. The reason is that the deviation of technical progress reflects factor input proportion. When considering human capital, the ratio of human capital to material capital is higher than that without feeling it. At present, China's education level is constantly improving. Two factors of human capital, labor force, and education level, increase simultaneously. Without human capital, the speed of capital-biased technical progress to neutral technological progress is higher.

Referring to the regression results, we directly obtain each index's average value in a period. However, equations (16), (17), (19) provide the method for calculating the index over the years. The calculation results are shown in the table below.

It can be seen from Table 3 that over the years, the relevant index of biased technical progress has been different from the average level among various periods. Except for the situation of D, the fluctuation of $\gamma_{K}$, $\gamma_{L}$ and $\gamma_{H}$ is obvious. In most years, the absolute value of $\gamma_{K}$ after considering human capital is more significant than that without considering human capital, and the relative relationship between $\gamma_{L}$ and $\gamma_{H}$ is just the opposite. If the fluctuation factors are removed, what will happen to each index? The following table shows the trend of each index after removing the fluctuation factors by the HP filter method.

**Table 3:** Various biased technical progress indices from 1986 to 2021.

Year	Without human capital			With human capital		
	$\gamma_{K}$	$\gamma_{L}$	D	$\gamma_{K}$	$\gamma_{H}$	D
1986	−0.09271	0.16406	0.09691	−0.10361	0.118521	0.071305
1987	−0.07774	0.31093	0.146687	−0.09065	0.272914	0.116705
1988	−0.02823	0.29048	0.120286	−0.03217	0.172266	0.065626
1989	−0.21863	0.09865	0.119744	−0.24662	−0.02898	0.069863
1990	0.096924	−0.09727	−0.07329	0.116783	−0.25134	−0.11817
1991	0.003594	0.29488	0.109936	6.75E-05	0.276171	0.08863
1992	0.141816	0.55912	0.157496	0.154831	0.571204	0.133658
1993	0.115497	0.57015	0.17159	0.127757	0.58945	0.148205
1994	−0.00321	0.43275	0.164532	−0.00132	0.470066	0.151316
1995	−0.08629	0.34927	0.164384	−0.09553	0.271924	0.117954
1996	−0.19169	0.38618	0.218094	−0.21759	0.320791	0.172823
1997	−0.1364	0.37669	0.193646	−0.15401	0.318099	0.151548
1998	−0.12534	0.35993	0.183143	−0.14137	0.300898	0.141971
1999	−0.20755	0.37293	0.219079	−0.23654	0.317412	0.177822
2000	−0.31139	0.35780	0.25256	−0.35391	0.284074	0.204794
2001	−0.13316	0.28255	0.156892	−0.15134	0.303951	0.146149
2002	−0.04555	0.38678	0.163165	−0.05311	0.415287	0.150359
2003	−0.09399	0.48537	0.21866	−0.11119	0.531288	0.206239
2004	−0.30199	0.67473	0.368625	−0.35509	0.747597	0.353967
2005	−0.04027	0.38529	0.160612	−0.04466	0.394483	0.140968
2006	−0.20186	0.50710	0.26757	−0.23082	0.461308	0.222175
2007	−0.10932	0.47140	0.219171	−0.12505	0.423405	0.176056
2008	0.296208	−0.0021	−0.11258	0.352335	−0.10213	−0.14589
2009	−0.08994	0.33300	0.159624	−0.10023	0.266066	0.117582
2010	−0.23694	0.49682	0.276927	−0.27229	0.455518	0.233628
2011	−0.15277	0.34949	0.189555	−0.17192	0.366449	0.172819
2012	−0.1206	0.27974	0.151093	−0.13336	0.26809	0.128868
2013	−0.33053	0.26887	0.226222	−0.37189	0.257237	0.201951
2014	−0.36203	0.25407	0.23252	−0.40665	0.248947	0.210448
2015	−0.23634	0.16744	0.152391	−0.26313	0.151731	0.133172
2016	−0.21021	0.34872	0.20586	−0.23677	0.33830	0.18432
2017	−0.15006	0.36476	0.19430	−0.16862	0.33947	0.16322
2018	−0.19887	0.29364	0.18418	−0.22296	0.27474	0.16024
2019	−0.23496	0.19373	0.19373	−0.26342	0.26665	0.17033
2020	−0.22203	0.20145	0.20145	−0.24950	0.29979	0.17764
2021	−0.21211	0.19732	0.19732	−0.23812	0.29455	0.17118

Results found that the absolute value of the trend after considering human capital is more significant than that without considering human capital, and the relative relationship between $\gamma_{L}$ and $\gamma_{H}$ is opposite (Table 4). Moreover, it is found that all types of biased technology progress indices exhibit an upward trend: capital productivity declines sharply, labor productivity (human capital) increases rapidly, and technical progress also accelerates towards capital. However, this trend has slowed down in recent years. Therefore, what about real technical progress? The following table shows the trend of capital productivity, labor productivity, and human capital productivity growth rates after HP filtering.

**Table 4:** Trends of various biased technical progress indices from 1986 to 2021.

Years	Without human capital			With human capital		
	$\gamma_{K}$_hp_trend	$\gamma_{L}$_hp_trend	D hp_trend	$\gamma_{K}$_hp_trend	$\gamma_{H}$_hp_trend	D hp_trend
1986	−0.09271	0.16406	0.09691	−0.10361	0.11852	0.07131
1987	−0.07774	0.31093	0.14669	−0.09065	0.27291	0.11671
1988	−0.02823	0.29048	0.12029	−0.03217	0.17227	0.06563
1989	−0.21863	0.09865	0.11974	−0.24662	−0.02898	0.06986
1990	0.09692	−0.09727	−0.07329	0.11678	−0.25134	−0.11817
1991	0.00359	0.29488	0.10994	0.00007	0.27617	0.08863
1992	0.14182	0.55912	0.15750	0.15483	0.57120	0.13366
1993	0.11550	0.57015	0.17159	0.12776	0.58945	0.14821
1994	−0.00321	0.43275	0.16453	−0.00132	0.47007	0.15132
1995	−0.08629	0.34927	0.16438	−0.09553	0.27192	0.11795
1996	−0.19169	0.38618	0.21809	−0.21759	0.32079	0.17282
1997	−0.13640	0.37669	0.19365	−0.15401	0.31810	0.15155
1998	−0.12534	0.35993	0.18314	−0.14137	0.30090	0.14197
1999	−0.20755	0.37293	0.21908	−0.23654	0.31741	0.17782
2000	−0.31139	0.35780	0.25256	−0.35391	0.28407	0.20479
2001	−0.13316	0.28255	0.15689	−0.15134	0.30395	0.14615
2002	−0.04555	0.38678	0.16317	−0.05311	0.41529	0.15036
2003	−0.09399	0.48537	0.21866	−0.11119	0.53129	0.20624
2004	−0.30199	0.67473	0.36863	−0.35509	0.74760	0.35397
2005	−0.04027	0.38529	0.16061	−0.04466	0.39448	0.14097
2006	−0.20186	0.50710	0.26757	−0.23082	0.46131	0.22218
2007	−0.10932	0.47140	0.21917	−0.12505	0.42341	0.17606
2008	0.29621	−0.00210	−0.11258	0.35234	−0.10213	−0.14589
2009	−0.08994	0.33300	0.15962	−0.10023	0.26607	0.11758
2010	−0.23694	0.49682	0.27693	−0.27229	0.45552	0.23363
2011	−0.15277	0.34949	0.18956	−0.17192	0.36645	0.17282
2012	−0.12060	0.27974	0.15109	−0.13336	0.26809	0.12887
2013	−0.33053	0.26887	0.22622	−0.37189	0.25724	0.20195
2014	−0.36203	0.25407	0.23252	−0.40665	0.24895	0.21045
2015	−0.23634	0.16744	0.15239	−0.26313	0.15173	0.13317
2016	−0.21021	0.34872	0.20586	−0.23677	0.33830	0.18432
2017	−0.15006	0.36476	0.19430	−0.16862	0.33947	0.16322
2018	−0.19887	0.29364	0.18418	−0.22296	0.27474	0.16024
2019	−0.23496	0.19373	0.19373	−0.26342	0.26665	0.17033
2020	−0.22203	0.20145	0.20145	−0.24950	0.29979	0.17764
2021	−0.21211	0.19732	0.19732	−0.23812	0.29455	0.17118

In Table 5, the actual capital productivity drops acceleratively. The real labor productivity and human capital productivity are constantly increasing. Moreover, the human capital productivity growth rate is lower than labor productivity. This identifies with various indices of biased technical progress obtained by theoretical analysis. The only inconsistency is that labor and human capital productivity growth rate has declined while the theoretical analysis is rising. Only in recent years has it refused. The growth rate of various indices calculated theoretically is the estimated value of a marginal quantity, while the actual situation is the average value.

**Table 5:** The trend of productivity growth (unit: percentage).

Year	Capital Productivity Growth Rate	Labor Productivity Growth Rate	Human Resources Productivity Growth Rate
1986	−1.10537	612.67338	29.88793
1987	−1.11858	86.21867	23.07465
1988	−1.13505	46.68094	18.89597
1989	−1.15664	32.31696	16.12813
1990	−1.18486	25.01122	14.20779
1991	−1.22436	20.67089	12.83256
1992	−1.28027	17.84804	11.82106
1993	−1.35592	15.89781	11.05849
1994	−1.4499	14.48907	10.47010
1995	−1.55688	13.43479	10.00758
1996	−1.67134	12.62045	9.63657
1997	−1.78871	11.97158	9.33163
1998	−1.90611	11.43724	9.07314
1999	−2.02167	10.98198	8.84564
2000	−2.13429	10.58028	8.63658
2001	−2.24433	10.21363	8.43631
2002	−2.3551	9.86799	8.23601
2003	−2.47072	9.53226	8.02884
2004	−2.59441	9.19856	7.81048
2005	−2.72799	8.86215	7.57908
2006	−2.87168	8.52036	7.33472
2007	−3.02512	8.17226	7.07883
2008	−3.18847	7.81853	6.81321
2009	−3.36123	7.46111	6.54026
2010	−3.54222	7.10313	6.26322
2011	−3.73001	6.74767	5.98476
2012	−3.92294	6.39860	5.70792
2013	−4.11938	6.06032	5.43652
2014	−4.3181	5.73732	5.17454
2015	−4.52119	5.43364	4.92606
2016	−4.72731	5.14197	4.68510
2017	−4.92343	4.86160	4.45012
2018	−5.13478	4.58766	4.22156
2019	−5.34772	4.32482	4.00523
2020	−5.57089	4.07453	3.79770
2021	−5.79678	3.83479	3.60108

## 5 Conclusion, implications, limitations and recommendations for future studies

### 5.1 Main findings and policy implications

This study aimed to estimate biased technical progress in China by considering heterogeneous human capital and labor force in a normalized supply-side system with a constant elasticity of substitution (CES) production function. The analysis employed a constrained seemingly unrelated regression (SUR) using provincial-level panel data from 1985 to 2021 and compared results with and without accounting for labor heterogeneity and human capital.

The main findings of the study are as follows. In China, the substitution elasticity of capital-labor (human capital) is between 0 and 1, indicating a complementary relationship between factors. The marginal productivity of capital continues to decline, while the marginal productivity of labor (human capital) increases, and technical progress is biased toward capital. Human capital increased the elasticity of substitution, but the increase was limited and did not change the complementary relationship between factors. The absolute values of γ and K were greater when considering human capital, indicating a faster decline in marginal capital productivity. The growth rate of marginal productivity of human capital was lower than that of the labor force. The index of biased technical progress considering human capital was smaller than without considering it, suggesting a decreased degree of capital-biased technical progress.

The faster decline in marginal productivity of capital after considering human capital can be attributed to the positive effect of human capital on economic growth. As human capital and the labor force increased, the contribution of capital to output decreased. Additionally, the total human capital increased, and its overall contribution increased. Consequently, the contribution of capital to output decreased not only due to the increase in capital itself but also due to the growth of human capital, resulting in a faster rate of decline compared to the case without considering human capital. The lower growth rate of marginal productivity of human capital compared to the labor force is due to the faster growth rate of human capital, which equals the sum of the growth rates of labor quantity and per capita education years. As both growth rates are positive, the growth rate of human capital is faster than that of the labor force. Marginal productivity is inversely proportional to the input scale, and after considering human capital, the marginal productivity growth rate is affected by the acceleration of the input growth rate.

The study found that human capital influences biased technical progress through two aspects: the elasticity of substitution and the marginal productivity growth rate. To adjust China's capital-biased technical progress, which has brought many problems, increasing human capital input is a feasible approach, especially when labor force growth is restricted by population growth. Furthermore, the impact of human capital on various growth rates of marginal productivity suggests that China's current capital investment is excessive. Although China's per capita material capital of the labor force is still lower compared to developed countries, the main difference lies in the lower content of science and technology across industries. China primarily relies on increasing material consumption and product quantity. To improve the marginal productivity of capital, in addition to moderately slowing down investment growth, China should adjust its industrial structure, increase science and technology content, and reduce the quantity and scale of production. This approach would also help in adjusting technical progress towards Hicks-neutral. The results suggest that increasing human capital investment could help adjust China's capital-biased technical progress, and moderating capital investment growth while increasing the technology content of industries could improve capital productivity. The results have important policy implications.

• To adjust the capital-biased direction of technical progress, increasing human capital investment is a feasible approach given constraints on labor force growth from population dynamics.
• The impact of human capital on factor productivity growth rates indicates current capital investment in China is excessive. While per capita capital stock for labor remains lower than in developed countries, the key difference is China's lower technology content across industries, with greater reliance on increased material consumption and product quantity.
• To improve capital productivity, in addition to moderating investment growth, adjusting the industrial structure to increase technology content and reduce scale and quantity could help shift technical progress toward Hicks-neutral.

Overall, the findings suggest a two-pronged approach of increasing human capital and technology content in production could rebalance China's factor productivity growth and technical progress.

### 5.2 Limitations and future research directions

While this study makes important contributions, it also has some limitations that suggest avenues for future research. First, the analysis relies on provincial-level data, which may obscure important within-province heterogeneity in factor inputs and technical progress. Future studies could use more disaggregated data, such as firm-level or county-level data, to provide a clearer picture of China's biased technical change. Second, the estimation framework, while an improvement over previous studies, is still relatively simple. It does not explicitly model the endogenous direction of technical change based on factor prices and supplies. Incorporating induced innovation effects could yield additional insights into a sustainable environment. Third, while standard in the literature, the measure of human capital is based solely on educational attainment. It does not account for the quality of education, on-the-job training, or other forms of skill acquisition. Developing more comprehensive measures of human capital could enrich the analysis. Fourth, the study does not directly examine the links between biased technical change and other key economic outcomes, such as income inequality, structural transformation, and trade patterns. Exploring these connections could shed light on the broader implications of China's technical progress. Finally, China's economy continues to evolve, and the nature and drivers of its technical change may shift as well. Ongoing research will be needed to track these dynamics and inform policy responses.

Despite these limitations, this study makes a valuable contribution to understanding the role of human capital and labor heterogeneity in shaping China's biased technical progress. It lays the groundwork for future research to deepen and extend this analysis, with important implications for China's long-term economic development.

## Has data associated with your study been deposited into a publicly available repository?

No.

## Data availability statement

Data will be available on request from the corresponding author.

## Funding statements

This study was supported by Major Humanities and Social Sciences Research Projects in Zhejiang Higher Education Institutions of China under Grant No.2023QN106; Key Research Center of Philosophy and Social Science of Zhejiang Provincial of China under Grant No.20JDZD072; 10.13039/501100004731Zhejiang Provincial Natural Science Foundation of China under Grant No.LGF19G030005; Project Research on the Mechanism, Path and Strategy of Artificial Intelligence Driving the High-Quality Development of Shandong Provincial Manufacturing Industry supported by 10.13039/501100007129Shandong Provincial Natural Science Foundation (ZR2022QG027) and the Taishan Young Scholar Program (No. tsqn202103070), the 10.13039/100012620Taishan Scholar Foundation of Shandong Province of China.

## Ethics approval and consent to participate

This research was reviewed and approved by the ethical committee for scientific research at the School of Economics, Shandong University of Technology. Verbal consent was taken from respondents to participate in the data collection process.

## Patient consent statement

Not applicable.

## Permission to reproduce material from other sources

Not applicable.

## Clinical trial registration

Not applicable.

## CRediT authorship contribution statement

**Xiuli Cui:** Software, Resources, Methodology, Investigation, Formal analysis, Data curation. **Ehsan Elahi:** Writing – original draft, Supervision, Funding acquisition, Conceptualization. **Bo Xu:** Data curation, Writing – review & editing. **Jiaxun Xing:** Methodology, Writing – review & editing. **Mohd Shuaib:** Writing – review & editing. **Zainab Khalid:** Writing – original draft, Conceptualization.

## Declaration of competing interest

The authors declare that they have no known competing financial interests or personal relationships that could have appeared to influence the work reported in this paper.

## Footnotes

2 *Data resources: Human Capital in China 2023, published by China Center for Human Capital and Labor Market Research of Central University of Finance and Economics*.

3 *Six categories of education are no school, primary school, junior middle school, senior high school, college, and university or above. Since the number of people receiving graduate education is relatively small, it has little impact on the average education year*.

4 *Source of data:*http://humancapital.cufe.edu.cn/rlzbzsxm.htm.
